# Procalcitonin in cerebrospinal fluid in meningitis: a prospective diagnostic study

**DOI:** 10.1002/brb3.545

**Published:** 2016-08-16

**Authors:** Imanda M. E. Alons, Rolf J. Verheul, Irma Kuipers, Korné Jellema, Marieke J. H. Wermer, Ale Algra, Gabriëlle Ponjee

**Affiliations:** ^1^ Department of NeurologyMCH WesteindeThe HagueThe Netherlands; ^2^ Department of Clinical chemistryLabWest/MCH WesteindeThe HagueThe Netherlands; ^3^ Department of NeurologyLUMCLeidenThe Netherlands; ^4^ Department of Clinical EpidemiologyLUMCLeidenThe Netherlands; ^5^ Department of Neurology and NeurosurgeryBrain Center Rudolph MagnusUMC UtrechtUtrechtThe Netherlands; ^6^ Julius Center for Health Sciences and Patient CareUMC UtrechtUtrechtThe Netherlands

**Keywords:** bacterial meningitis, cerebrospinal fluid, diagnostic marker, external ventricular drain, meningitis, neurosurgical intervention, procalcitonin

## Abstract

**Objectives:**

Bacterial meningitis is a severe but treatable condition. Clinical symptoms may be ambiguous and current diagnostics lack sensitivity and specificity, complicating diagnosis. Procalcitonin (PCT) is a protein that is elevated in serum in bacterial infection. We aimed to assess the value of PCT in cerebrospinal fluid (CSF) in the diagnosis of bacterial meningitis.

**Methods:**

We included patients with bacterial meningitis, both community acquired and post neurosurgery. We included two comparison groups: patients with viral meningitis and patients who underwent lumbar punctures for noninfectious indications. We calculated mean differences and 95% confidence intervals of procalcitonin in CSF and plasma in patients with and without bacterial meningitis.

**Results:**

Average PCT concentrations in CSF were 0.60 ng mL^−1^ (95% CI: 0.29–0.92) in the bacterial meningitis group (*n* = 26), 0.81 (95% CI: 0.33–1.28) in community‐acquired meningitis (*n* = 16) and 0.28 (95% CI: 0.10–0.45) in postneurosurgical meningitis (*n* = 10), 0.10 ng mL^−1^ (95% CI: 0.08–0.12) in the viral meningitis group (*n* = 14) and 0.08 ng mL^−1^ (95% CI: 0.06–0.09) in the noninfectious group (*n* = 14). Mean difference of PCT‐CSF between patients with community‐acquired bacterial meningitis and with viral meningitis was 0.71 ng mL^−1^ (95% CI: 0.17–1.25) and 0.73 ng mL^−1^ (95% CI: 0.19–1.27) for community‐acquired bacterial meningitis versus the noninfectious group. The median PCT CSF: plasma ratio was 5.18 in postneurosurgical and 0.18 in community‐acquired meningitis (IQR 4.69 vs. 0.28).

**Conclusion:**

Procalcitonin in CSF was significantly higher in patients with bacterial meningitis when compared with patients with viral or no meningitis. PCT in CSF may be a valuable marker in diagnosing bacterial meningitis, and could become especially useful in patients after neurosurgery.

## 1 Introduction

Bacterial meningitis is a life threatening but, treatable condition with high morbidity (20%) and mortality (15%) (de Gans & van de Beek, 2002). The gold standard in diagnosing bacterial meningitis is by demonstrating the presence of bacteria in cerebrospinal fluid (CSF) samples via gram staining or CSF cultures. In the acute setting, however, bacterial cultures take too much time to be used in the decision whether or not to start antibacterial treatment. Polynuclear pleocytosis combined with raised CSF protein and lowered CSF glucose indicate bacterial meningitis, but these findings are not sensitive and may not be present in 12% of patients (van de Beek, Drake, & Tunkel, 2010; van de Beek et al., 2004; Durand et al., 1993). These parameters are even more unreliable in patients with an extraventricular drain (EVD) after subarachnoid‐ or intracranial hemorrhage as intrathecal plasma leakage causes raised erythrocytes and leukocytes counts (Schade et al., 2006). An additional diagnostic marker to distinguish bacterial meningitis from aseptic, viral, or no meningitis would therefore be valuable.

Procalcitonin (PCT) is a protein that can be released by parenchymal cells in the presence of endotoxins or cytokines like interleukin‐6 and tumor necrosis factor‐α. PCT is raised strongly during bacterial inflammation, but not or only marginally elevated in viral or noninfectious inflammatory reactions (Karzai, Oberhoffer, Meier‐Hellman, & Reinhart, 1997; Meisner, 2000). Whether it is locally produced in the central nervous system is a matter of debate, but calcitonin messenger RNA has been isolated in hamster brain tissue suggesting this possibility (Müller et al., 2001). A recent study showed that procalcitonin is produced by trigeminal glia cells in response to inflammation, making local production in the central nervous system more likely (Raddant & Russo, 2014).

Availability of the PCT‐assay on standard immunochemistry platforms allows turnaround times within an hour, making the test suitable in an acute setting. PCT has been proven to be valuable in differentiating between bacterial and viral meningitis when determined in serum (Dubos et al., 2008; Gendrel et al., 1997; Viallon et al., 2011). However, the usefulness of PCT in serum of patients with bacterial meningitis after neurosurgical intervention is limited (Choi & Choi, 2013). Previous studies of PCT measurement in CSF have shown conflicting outcomes. Two studies showed significantly higher PCT concentrations in CSF in patients with bacterial meningitis compared with tick‐borne encephalitis or viral meningitis (Jereb, Muzlovic, Hojker, & Strle, 2001; Konstantinidis et al., 2015). Others have suggested that the level of PCT in CSF did not differ between bacterial and viral meningitis (Shimetani, Shimetani, & Mori, 2001). Methodological factors may explain these conflicting findings. As the analytical performance of the PCT assay has improved since these studies, renewed investigation is warranted.

The aim of this study is to investigate whether there PCT levels in CSF in patients with bacterial meningitis are elevated and whether this could be a potential marker to differentiate between the presence or absence of bacterial meningitis.

## 2 Materials and Methods

### 2.1 Patients

From September 2012 to February 2015, we prospectively included adult patients (>18 years) who underwent a lumbar puncture on clinical suspicion of bacterial meningitis. Patients presented to the emergency room, were either admitted or were seen as inpatients in our secondary teaching hospital. We aimed to study consecutive patients, however, a small number of patients were missed when seen during on‐call hours.

The study protocol was evaluated and approved by the local ethics committee. The ethics committee waived the need for consent to participate as PCT was determined in already available CSF and plasma samples (remnants) and no additional sample volumes were taken for this study. Patients were investigated and treated according to current clinical practice and were not personally subjected to additional testing or questionnaires.

We divided patients into three groups depending on the outcome of standard CSF examination. First, a bacterial meningitis group, both community‐acquired (CAM) or after neurosurgical procedure (PNM), second, a comparison group with viral meningitis, and finally a comparison group who underwent lumbar puncture for noninfectious reasons.

Patients were diagnosed with bacterial meningitis if CSF testing showed polynuclear pleocytosis with leukocyte count >2,000 × 10^6^ per liter CSF leukocytes, raised CSF protein >2.2 g L^−1^, lowered CSF glucose <1.9 mmol L^−1^ or a CSF:plasma glucose ratio <0.23. Positive CSF culture, if available, was used as diagnostic gold standard. These conditions have been proven to be 88%–99% accurate individual predictors of bacterial meningitis (van de Beek et al., 2010, 2004). Our bacterial meningitis group consisted of patients with community‐acquired meningitis (CAM) and patients with postneurosurgical intervention meningitis (PNM). The interventions in the PNM group consisted of placement of an EVD or craniotomy. By using the same CSF chemistry criteria for the PNM group, we aimed to collect not only a homogenous group with bacterial meningitis but also to exclude patients with aseptic meningitis. Second, we hoped to evaluate the differences in PCT outcome between CAM and PNM groups with similar CSF chemistry.

The first comparison group consisted of patients with viral meningitis. A positive PCR was used as gold standard where available. Patients were diagnosed with viral meningitis if CSF testing showed mononuclear pleocytosis with leukocyte count >5 × 10^6^ per liter, but normal glucose and possibly raised protein.

The second comparison group consisted of patients free of infection. The selection criteria for this group were that patients were free of fever (T < 38°C) and the indication for the LP was unrelated to excluding infectious disease (e.g., hemorrhage in acute headache), additionally normal plasma C‐reactive protein (<5 mg L^−1^), normal CSF leukocyte count (<5 × 10^6^ per liter) and normal CSF glucose (>1.9 mmol L^−1^) and protein (<2.2 L) were mandatory inclusion criteria for this group.

We collected data on clinical characteristics, such as presence of headache, fever, depressed level of consciousness, nuchal rigidity, and whether bacterial cultures were positive and with which pathogen.

### 2.2 Sample handling and assays

Lumbar punctures were performed and plasma was obtained before the start of treatment. PCT was determined from the same CSF that prompted a diagnosis of bacterial meningitis or not. The PCT results were not visible for clinicians during diagnosis or treatment. CSF samples obtained from the lumbar puncture were taken to the in‐house clinical laboratory within 20 min. The least blood‐stained specimens were used for analysis. After a visual inspection and cell‐count, samples were centrifuged for 5 min at 1,250 g, within 1 hr after lumbar puncture. Total protein in CSF was measured turbidimetrically after adding benzethoniumchloride, glucose levels were determined with the hexokinase method, and CRP by immunoturbidimetry. Plasma and CSF procalcitonin were measured with the BRAHMS Elecsys two‐step immunoassay. If direct measurement of PCT was not possible, samples (CSF and/or plasma) were stored at −20°C within 24 hr. The functional sensitivity of the PCT assay was <0.06 ng mL^−1^, with an analytical sensitivity of <0.02 ng mL^−1^. There is a reported variation coefficient of <10% at a concentration of 0.04 ng mL^−1^ in plasma (Lloyd & Kuyl, 2012). All automated immunochemistry assays were done on a Modular analyser (Roche, Almere, The Netherlands). Standard CSF chemistry and PCT determination were evaluated by the hospital clinical chemist.

Reported reference plasma PCT concentration in healthy adults is <0.10 ng mL^−1^. Reference values for PCT in CSF are unknown (Lloyd & Kuyl, 2012). Plasma PCT was not determined in the two comparison groups.

### 2.3 Statistical analysis

We calculated the mean differences in PCT levels between patients with and without bacterial meningitis, as well as accompanying 95% confidence intervals (CI). If the 95% confidence interval for the difference does not contain the value 0, the difference is statistically significant and *p* < .05. For comparison of PCT in plasma and PCT ratios in CSF versus plasma between groups, we used the Mann–Whitney *U* test because the data of these variables were not normally distributed. For these data, we gave medians and interquartile ranges (IQR's). To assess the discriminative property for PCT in CSF, we made ROC curves and calculated the area under the curve comparing patient groups with an infection (bacterial meningitis, both CAM and PNM, and viral meningitis) with the patients without an infection.

## 3 Results

We included 26 patients with bacterial meningitis; 16 patients with community‐acquired and 10 patients with bacterial meningitis after neurosurgical intervention. We excluded four patients with bacterial meningitis who clinically were treated as such, but did not meet the criteria set in advance for CSF diagnosis. Of the PNM patients, four had an EVD and six a craniotomy. There were 13 positive CSF cultures among the 26 patients; three Pneumococcus *pneumonia*, three Staphylococcus *epidermidis*, two Neisseria *meningitides*, one Heamophilus *influenza*, one Escherichia *coli*, one Streptoccus *mitis*, one Streptococcus *pneumoniae*, and one Listeria *monocytogenes*. The CSF cultures of the remaining patients were negative. In the comparison groups, we included 14 patients with viral meningitis and 14 patients without infection. In patients with viral meningitis, PCR was positive for enterovirus in three cases, herpes simplex‐2 in one case, and varicella‐zoster in the last case. In these groups, no patients were excluded. Patient characteristics and results are shown in Tables 1 and 2.

**Table 1:** Patient characteristics

	Bacterial meningitis Entire group (*n* = 26)	Bacterial CAM (*n* = 16)	Bacterial PNM (*n* = 10)	Viral meningitis (*n* = 14)	Noninfectious (*n* = 14)
Age (years)	62 (SD 16)	60 (SD 18.6)	64 (SD 12)	34 (SD 9)	44 (SD 14)
Range	20–84	20–84	51–83	20–50	22–71
Sex (male)	19 (73%)	12 (75%)	7 (70%)	6 (43%)	4 (29%)
Fever	18 (69%)	12 (75%)	6 (60%)	6 (43%)	0
Ave CRP mg L^−1^ (SD)	121 (102)	178 (88)	36 (44)	14 (28)	1.5 (1.2)
Nuchal rigidity	14 (50%)	10 (63%)	4 (40%)	3 (21%)	0
Glasgow coma score ave	11	11	12	15	15
Headache	19 (73%)	12 (75%)	7(70%)	14 (100%)	11 (78%)
Culture positive	13 (50%)	10 (63%)	3 (33%)	5 (36%)	0

CAM, community‐acquired meningitis; PNM, postneurosurgical meningitis.

**Table 2:** Results of cell counts, glucose, protein and PCT in CSF and PCT in plasma per group

	Bacterial meningitis (*n* = 26)	CAM (*n* = 16)	PNM (*n* = 10)	Viral meningitis (*n* = 14)	Non‐infectious (*n* = 14)
CSF leukocyte count × 10^6^ per liter ave	5,998	7,551	3,514	267	1
Polynuclear cells × 10^6^ per liter ave	5,589	7,428	2,832	28	0.1
Mononuclear cells × 10^6^ per liter ave	616	576	677	239	0.7
Erythrocytes × 10^6^ per liter ave	23,649	12,892	408,597	180	287
CSF glucose mmol L^−1^ ave	1.6	1.0	2.6	3.5	3.4
CSF protein g L^−1^ ave	3.3	3.9	2.4	1	0.4
PCT in CSF ng mL^−1^Average (95% CI)	0.61 (0.29–0.90)	0.81 (0.31–1.31)	0.29 (0.10–0.45)	0.10 (0.08–0.12)	0.08 (0.05–0.09)
PCT in plasma ng mL^−1^Median (IQR)	0.5 (4.36)	1.28 (6.82)	0.05 (0.08)	0.02 (0.02)	–
PCT ratio CSF:plasmaMedian (IQR)	0.86 (2.79)	0.18 (0.27)	5.18 (4.69)	3.00 (1.38)	–
Mean difference PCT in CSF versus non infectious (95% CI)	0.74 ng mL^−1^ (0.20–1.28)	0.73 ng mL^−1^ (0.20–1.27)	0.21 ng mL^−1^ (0.05–0.37)	0.30 ng mL^−1^ (−0.001 to 0.05)	–
Mean difference PCT in CSF versus Viral meningitis (95% CI)	0.73 ng mL^−1^ (0.19–1.27)	0.71 ng mL^−1^ (0.18–1.25)	0.18 ng mL^−1^ (0.02–0.34)	–	–

CAM, community‐acquired meningitis; PNM, postneurosurgical meningitis; CSF, cerebrospinal fluid; PCT, procalcitonin.

**Figure 1:**
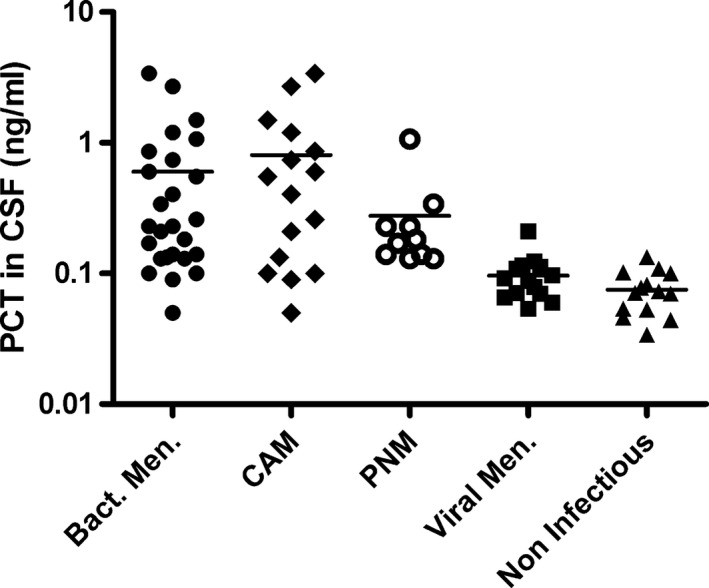
Scatter plots of procalcitonin in cerebrospinal fluid in the studied groups. Horizontal bar represents the mean value of a group, logarithmic scale

The average PCT in CSF in the group of bacterial meningitis patients was 0.61 ng mL^−1^ (95% CI: 0.27–0.93). The PCT in CSF in the group of patients with bacterial meningitis was significantly higher when compared with viral meningitis and the group without infection PCT in CSF (Table 2). PCT in CSF was not significantly different between CAM or PNM patients (mean difference 0.53 ng mL^−1^; 95% CI: −0.13 to 1.19). The PCT in CSF of the PNM patients was significantly higher than the viral meningitis group and the group without infection (Fig. 1, Table 2).

**Figure 2:**
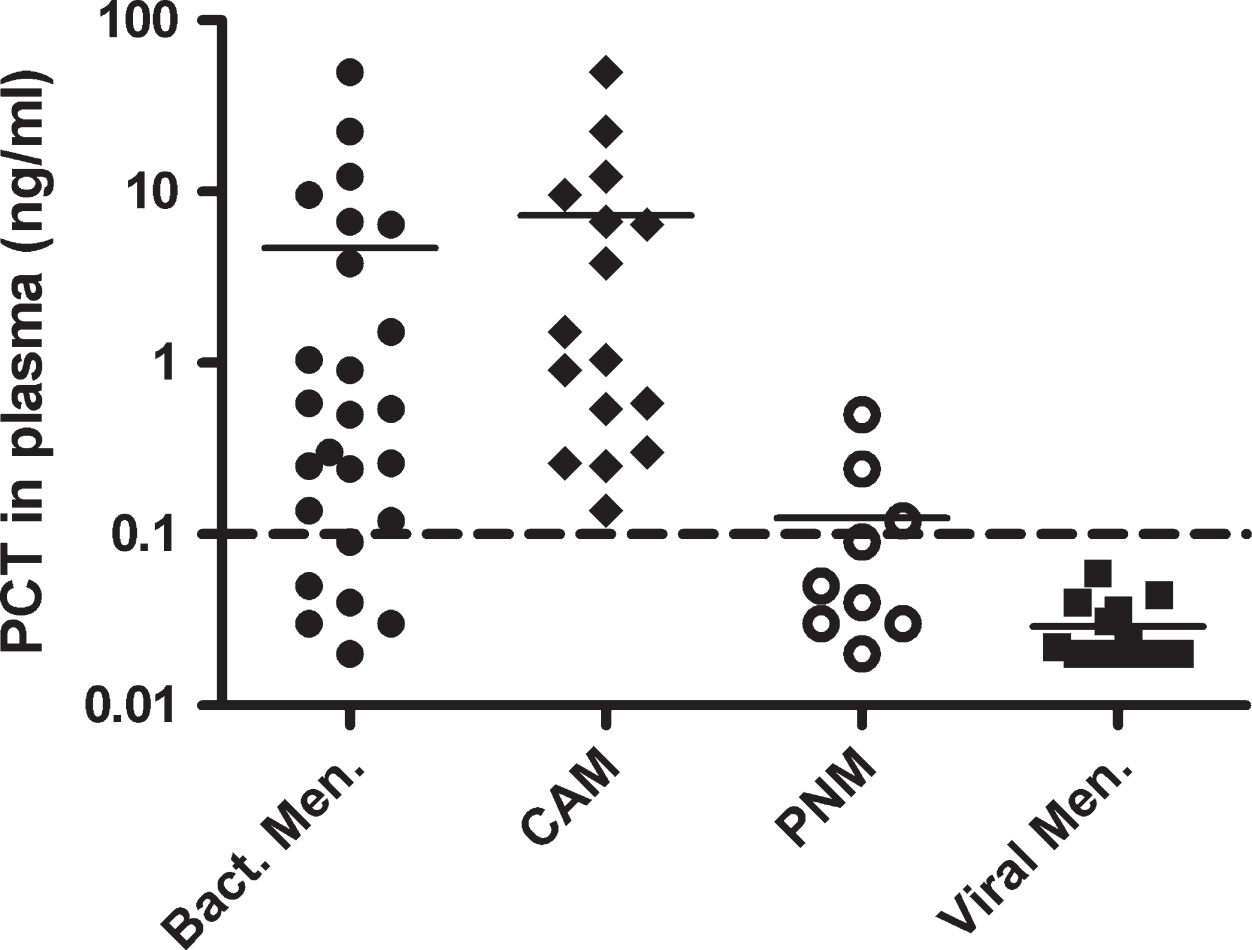
Scatter plots of procalcitonin in plasma in the studied groups. Horizontal bar represents the mean value of a group, logarithmic scale. The dotted line represents procalcitonin normal value in plasma

The median PCT in plasma of the entire bacterial meningitis group was 0.50 ng mL^−1^ (IQR 4.36). The median levels of PCT in plasma showed a statistically significant difference between the bacterial and viral meningitis groups (*p* =< .001). The median plasma PCT in the PNM group of 0.05 ng mL^−1^ (IQR 0.08) was significantly lower than that in the CAM group of 1.28 ng mL^−1^ (IQR 6.82) (*p* =< .001) (Fig. 2). Five out of 10 patients (50%) in the PNM group had plasma PCT levels within the reported reference range of <0.1 ng mL^−1^.

**Figure 3:**
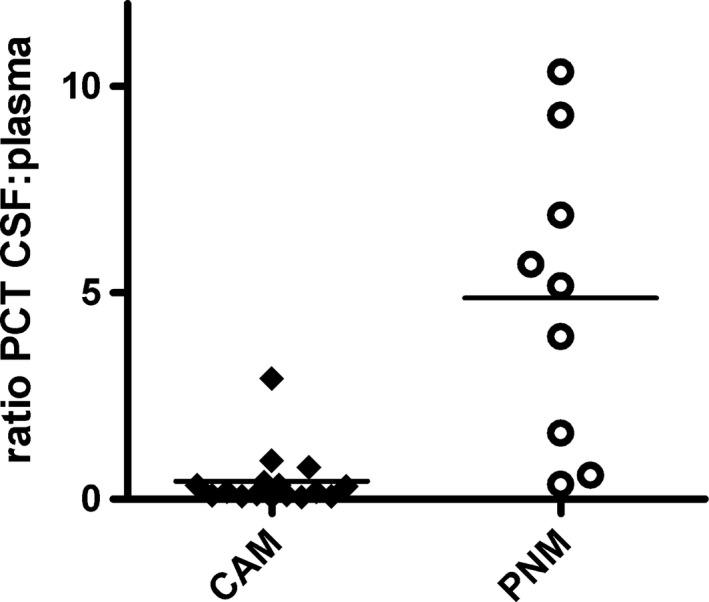
Scatter plots of procalcitonin plasma: cerebrospinal fluid ratio. Horizontal bar represents the mean value of a group, logarithmic scale

The PCT CSF: plasma ratio was significantly higher in PNM patients (median 5.18, IQR 4.69) than in patients with CAM (median 0.18, IQR 0.27) (*p* < .001), due to a higher PCT in CSF in comparison with PCT plasma levels in PNM patients (Fig. 3). We also compared these ratios between patients with low and high erythrocyte counts (<40 and ≥2,000 × 10^6^ per liter erytrocytes) in order to ascertain whether outcome was influenced by traumatic lumbar puncture both in patients with CAM and PNM. In the CAM group, nine patients had ≥2,000 × 10^6^ per liter erythrocytes in CSF and five CAM patients had an erythrocyte count <40 × 10^6^ per liter. The PCT CSF:plasma ratio in these groups was not significantly different (ery's <40 median 0.18, IQR 0.31, ery's >2,000 median 0.33, IQR 0.25) (*p* = 1). In PNM, eight patients had ≥2,000 × 10^6^ per liter erythrocytes in the CSF. In this group, the median PCT CSF: plasma ratio was 5.18 (IQR 3.18). Only one patient had <40 × 10^6^ per liter erythrocytes in his CSF with a CSF: plasma ratio of 1.6. The final PNM patient had 597 × 106 per liter erythrocytes and a PCT CSF:plasma ratio of 9.3.

We also compared PCT levels in patients diagnosed with bacterial meningitis with and without positive bacterial CSF cultures. The average PCT in CSF of patients with a positive culture was 0.82 ng mL^−1^ (SD 1.03, *N* = 13). The average PCT in CSF of patients with a negative culture was 0.41 ng mL^−1^ (SD 0.35, *N* = 13). The difference between these levels was not statistically significant (mean difference 0.41 ng mL^−1^; 95% CI: −0.18 to 1.00). In the PNM group, four patients received an external ventricular drain and six underwent a craniotomy. Statistical analysis showed no effect of type of neurological surgery on PCT values (mean difference 0.06; 95% CI: −0.39 to 0.51). PCT in plasma was not significantly lower in patients after EVD than in patients after craniotomy (mean difference 0.16; 95% CI: −0.06 to 0.38). However, the confidence interval is wide due to the small patient subgroup. In PNM, three CSF cultures were positive which were all found in the craniotomy group.

**Figure 4:**
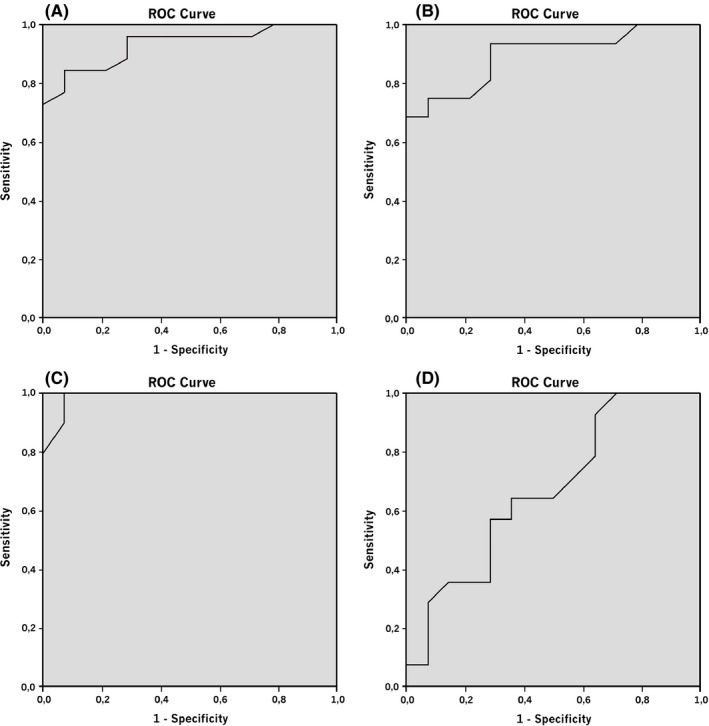
Receiver–operator curves for Procalcitonin in cerebrospinal fluid compared to noninfectious patients for (A) all bacterial meningitis patients, AUC 0.93, (B) community‐acquired meningitis. AUC 0.90, (C) post neurosurgical meningitis, AUC 0.98, (D) viral meningitis, AUC 0.67

The receiver–operator curve (ROC) for PCT in CSF of patients with bacterial meningitis versus the patients without an infection had an area under the curve (AUC) of 0.93 (95% CI: 0.86–1.00). The AUC of the ROC for the CAM versus the group of patients without an infection was 0.90 (95% CI: 0.78–1.00) and PNM versus group of patients without an infection was 0.99 (95% CI: 0.96–1.00). The AUC of the ROC for viral meningitis versus the group of patients without an infection was 0.67 (95% CI: 0.47–0.87) (Fig. 4).

In order to be able to calculate sensitivity specificity and positive and predictive value, we chose a cutoff value for PCT in CSF based on our reference groups. We arbitrarily chose a cutoff value for PCT in CSF of >0.9 ng mL^−1^. This is the upper limit of the 95% CI of PCT in CSF in the noninfectious group. With this cutoff value, we found a sensitivity of 92% (95% CI: 75%–99%) and a specificity of 68% (95% CI: 48%–84%). Positive predictive value is 73% (95% CI: 55%–87%) and negative predictive value is 90% (95% CI: 70%–99%).

## 4 Discussion

Our results show that in our patient population, PCT in CSF is significantly higher in patients with bacterial meningitis when compared to patients from the two comparison groups: viral meningitis and the group of patients without an infection. This counts for patients with community‐acquired meningitis as well as patients with meningitis after a neurosurgical procedure.

Procalcitonin in plasma of patients with bacterial meningitis was significantly higher than that of patients with viral meningitis. But, PCT in plasma of the PNM group was not significantly higher than in the viral meningitis comparison group. For this subgroup, PCT in plasma seems less sensitive than PCT in CSF. This finding corresponds with an earlier study that evaluated the use of PCT in serum of postneurosurgical patients and found that serum sensitivity of PCT is low for diagnosing bacterial meningitis (Choi & Choi, 2013).

The findings above raise the question whether there is intrathecal production of PCT. Due to its large molecular structure (13 kDa) and being a protein, it is unlikely that PCT passes the blood–brain barrier in healthy adults (Maruna, Nedelníková, & Gürlich, 2000). However, in meningitis, the blood–brain barrier may be compromised due to infectious processes. Similarly, traumatic lumbar puncture may confound results by plasma leakage. Most of our bacterial meningitis samples had high erythrocyte counts (see Table 2). However, when patients with low or high erythrocyte counts with CAM and PNM were grouped and compared, PCT ratios were the same irrespective of erythrocyte count. This makes it highly unlikely that the elevated PCT in CSF originates from leakage of PCT plasma through the blood–brain barrier or traumatic lumbar puncture.

Strikingly, the CSF:plasma ratio of PCT was significantly higher in the PNM group compared with the CAM patients. This indicates a much stronger increase of PCT in CSF than in plasma in the PNM group. Moreover, most plasma PCT levels in the PNM group were within the reported reference range of <0.10 ng mL^−1^. We hypothesize this skewed distribution in favor of PCT in CSF in the PNM group may be due to a direct bacterial port of entry after neurosurgery and the lower level of systemic infection in these patients. We surmise PCT levels may only become raised in blood if bacterial meningitis is accompanied by systemic infection or sepsis, as is the case in most CAM patients. For patients with PNM determining PCT in CSF may become useful as a diagnostic marker.

Our study has limitations. First, study size is relatively small, limiting the power of the study. However, despite the small number of patients, we still found a significant difference between groups, with confidence intervals that are sufficiently narrow to allow precise determination of between group differences. Second, we could not prove the presence of meningitis in all patients by means of a positive CSF culture, resulting in potential bias due to misclassification. Since only 44% of patients have all characteristics of bacterial meningitis (van de Beek et al., 2010), we may have included patients in the wrong group. To diminish this possibly important methodological shortcoming, we used international CSF diagnostic values which are individual positive predictors in 88%–99% (van de Beek et al., 2010, 2004). In choosing these parameters, we also hoped to bypass the chance of diagnosing aseptic meningitis as bacterial meningitis. The downside of using these set parameters was that we could not calculate the added value of PCT against the conventional diagnostic markers. The calculation of an ROC curve of PCT in CSF versus leukocyte count for instance was not possible.

We calculated whether there was a difference in PCT levels in CSF in patients diagnosed with bacterial meningitis with a positive and negative bacterial CSF culture and found that the difference is not statistically significant. This supports our correct selection of patients for the bacterial meningitis group based on CSF chemistry.

Finally, there are no reference values for PCT in CSF available. By including a group with major headache as complaint and no CSF abnormalities, we aimed to include a “best‐available” reference group for CSF PCT. We arbitrarily determined a theoretical reference value based on the upper limit of the 95% CI of PCT in CSF for the noninfectious group. With this value, we calculated sensitivity, specificity, negative predictive, and positive predictive value for PCT in CST. These results show a good sensitivity and negative predictive value, but need to been seen only as a measure for easier interpretation of the outcomes; they cannot be extrapolated for clinical use. Future research will be needed to determine validated reference values.

As we have measured PCT in CSF at one point in time only, we do not know at what moment after infection peak levels are reached or when PCT levels start to decrease again. This is a highly interesting and clinically relevant issue and we are presently performing a follow‐up study in which daily PCT levels are measured in patients with an external ventricular drain.

Our results show that PCT in CSF could become a useful marker in diagnosing bacterial meningitis. This may in particular be the case in patients with suspected meningitis after neurosurgical intervention (in contrast to plasma PCT). However, as the PNM group was limited, this conclusion must be tentative. To the best of our knowledge, this is the first article including results of procalcitonin both in plasma and in CSF in a varied range of patients groups. An evaluation of PCT CSF:plasma ratios in patients with bacterial meningitis has not been done before. A recent study confirmed our results in CSF, but plasma levels were not included and therefore a direct comparison of PCT in CSF and plasma was lacking (Konstantinidis et al., 2015). The use of PCT in CSF in determining the need for and possibly duration of antibiotic treatment was beyond the scope of this study, but we hope our data will open the path to research in that direction. Further research on this subject is needed to assess the usefulness of this marker in clinical practice.

We found that PCT is significantly raised in CSF in patients with bacterial meningitis when compared to patients with viral or no meningitis. PCT in CSF may become a useful biomarker in this patient group and especially in patient after neurosurgery.

## Funding Information

No funding information provided.

## Conflict of Interest

The authors declare that they have no competing interests.
